# Post-divorce growth: a mixed-methods investigation of how post-traumatic stress shapes growth after divorce

**DOI:** 10.3389/fpsyg.2026.1850508

**Published:** 2026-05-28

**Authors:** Mustafa Selim Altinisik

**Affiliations:** Department of Educational Sciences, Division of Psychological Counseling and Guidance, Faculty of Education, Ondokuz Mayıs University, Samsun, Türkiye

**Keywords:** divorce, mixed-methods design, post-divorce growth, post-traumatic stress, post-traumatic growth, social support

## Abstract

**Introduction:**

This study aimed to examine the relationship between post-traumatic stress (PTS) and post-traumatic growth (PTG) after divorce among divorced individuals and to identify factors influencing growth through quantitative and qualitative findings.

**Methods:**

A mixed-methods design was employed in two phases. In the first phase, data were collected from 489 participants using a Personal Information Form, the PTSD Checklist for DSM-5 (PCL-5), and the Posttraumatic Growth Inventory (PTGI). In the second phase, four groups were formed based on high and low scores, and qualitative data were obtained from 16 participants through semi-structured interviews.

**Results:**

Quantitative findings showed that PTS was negatively associated with PTG, *r* = −0.32, *p* < 0.001, and statistically predicted PTG, explaining 10% of its variance, *R*^2^ = 0.10. In addition, PTG differed significantly by age and duration of marriage, whereas no significant differences were found by parenthood status or receipt of psychological support. Qualitative findings revealed that facilitating factors included hope, social support, spiritual coping, and cognitive restructuring, whereas inhibiting factors involved rumination, economic difficulties, social pressure, and negative emotions.

**Discussion:**

Overall, the findings suggest that post-traumatic growth after divorce is associated with the interaction of individual and contextual factors, highlighting the importance of psychosocial support in the growth process.

## 1 Introduction

Marriage is a significant union that fulfills individuals’ emotional, social, and economic needs and is grounded in trust, belonging, intimacy, and shared life goals (Maulana et al., 2025; Light and Fitzsimons, 2014; Anderson, 2013; Barber, 1974). It also plays a central role in promoting both individual and societal wellbeing (Sillah, 2014; Waite, 1995; Gove et al., 1990). Despite its importance, a substantial proportion of marriages end in divorce, highlighting a growing global concern (Andersson et al., 2025; Sheykhi, 2020). Recent data from the Turkish Statistical Institute indicate a notable increase in divorce rates, with nearly one in three marriages ending in divorce in 2025 (Türkiye İstatistik Kurumu, 2026).

Various factors contribute to divorce, including communication problems, domestic violence, emotional disengagement, conflicts over responsibilities, and mismatched expectations (Ottakkam Thodukayil et al., 2026; Sunte, 2025; Nisa, 2021; Hawkins et al., 2012; Gigy and Kelly, 1993). These findings suggest that divorce is not merely a legal event but a complex life transition that involves significant psychological and social adjustments (Cohen and Finzi-Dottan, 2012). Following divorce, individuals often experience disruptions in daily routines, roles, and future plans (Brown et al., 2023; Glick, 1984). This period is frequently accompanied by intense emotional responses such as sadness, loneliness, anger, guilt, rejection, and uncertainty (Koren et al., 2025; Imran, 2023; van Tilburg et al., 2015; Frisby et al., 2012). Moreover, changes in social networks, economic challenges, and shifts in parenting responsibilities may further intensify the negative impact of divorce (Muhammad et al., 2025; Mikucki-Enyart et al., 2018; Terhell et al., 2004).

Although the impact of divorce varies across individuals, it is often perceived as a traumatic life event and may lead to symptoms of post-traumatic stress disorder (PTSD) (Taylor, 2004). Experiences such as intense conflict, infidelity, violence, abandonment, or separation from children can trigger PTSD-related responses, including re-experiencing, avoidance, and hyperarousal (Ngambi et al., 2023; Fischel-Wolovick, 2018; Kitching, 2008; Chung et al., 2003; Morris and West, 2001).

Despite its traumatic nature, divorce does not necessarily result in only negative outcomes. Traumatic experiences may also facilitate positive psychological changes, reflecting the transformative potential of adversity (Ceylan and Kışlak, 2024; Dreman, 1991). This process is conceptualized as post-traumatic growth (PTG), which involves positive changes such as increased personal strength, a renewed sense of meaning in life, and deeper interpersonal relationships (Jayawickreme et al., 2021). PTG should be distinguished from resilience and recovery. Recovery refers to a return to pre-trauma functioning, whereas resilience reflects the capacity to maintain or regain adaptive functioning despite adversity (Joseph and Linley, 2005; Bonanno, 2004). PTG, however, refers to positive psychological changes that go beyond previous levels of functioning and emerge through the struggle with traumatic or highly stressful experiences (Tedeschi and Calhoun, 2004). PTG may also manifest through enhanced appreciation of life, recognition of new possibilities, and spiritual development (Joseph et al., 2012). In this context, although divorce represents a challenging life transition, it may also provide an opportunity for personal transformation and growth in some individuals (Badri et al., 2025).

In the present study, post-divorce growth is conceptualized as a divorce-specific manifestation of post-traumatic growth rather than as a separate construct. It refers to positive psychological changes that may emerge as individuals struggle with divorce-related stressors. Accordingly, the term post-traumatic growth (PTG) is used throughout the manuscript when referring to the theoretical construct and measurement, whereas post-divorce growth is used only to emphasize the divorce-related context of the study.

The relationship between post-traumatic stress (PTS) and post-traumatic growth (PTG) is complex rather than linear. While some studies suggest that higher levels of stress may hinder growth, others indicate that moderate levels of stress can promote growth by encouraging individuals to re-evaluate their lives (Liu et al., 2017; Shakespeare-Finch and Lurie-Beck, 2014). This suggests that the association between PTS and PTG may vary across levels of stress and is not universally consistent (Dell’Osso et al., 2022). Accordingly, PTG appears to depend not only on the intensity of stress but also on how individuals cognitively process and make meaning of their experiences (Rezapour, 2024; Slanbekova et al., 2017).

In this context, post-traumatic growth cannot be explained solely by stress levels. Psychosocial factors such as social support, economic conditions, family and societal attitudes, stigma, coping strategies, and spiritual resources may play a critical role in shaping post-divorce adjustment (Muhammad et al., 2025; Wallerstein and Kelly, 2013; Terhell et al., 2004; Hanson et al., 1998). These processes are further influenced by individual characteristics and meaning-making styles, leading to diverse outcomes. Thus, PTG should be considered a multidimensional process emerging from the interaction of individual and contextual factors.

Existing research has largely examined PTS and PTG as separate variables using quantitative approaches, focusing primarily on the direction and strength of their association. Studies specifically addressing divorce are limited and often focus on pre-divorce traumatic experiences (Slanbekova et al., 2014). Moreover, previous research has been insufficient in explaining the psychosocial mechanisms underlying this relationship and individual differences in post-divorce experiences. Given the multidimensional nature of divorce, a deeper exploration of factors that facilitate or hinder growth is essential. In addition, changing social and cultural conditions may influence divorce-related experiences, highlighting the need for updated and context-sensitive research.

Accordingly, the present study aimed not only to examine the relationship between PTS and PTG among divorced individuals but also to identify the psychosocial mechanisms shaping this relationship. A mixed-methods design was employed. In the quantitative phase, relationships between variables were analyzed, whereas in the qualitative phase, the processes facilitating and hindering growth were explored in depth among selected participant profiles. The quantitative hypotheses tested were: (H1) gender differences in PTG; (H2) age differences in PTG; (H3) differences based on parenthood status; (H4) differences based on duration of marriage; (H5) differences based on receiving psychological support; (H6) a negative association between PTS and PTG; and (H7) PTS as a negative predictor of PTG. The qualitative component addressed the following research questions: What factors facilitate post-traumatic growth after divorce? What factors hinder post-traumatic growth after divorce? How do experiences differ based on levels of stress and growth?

## 2 Method

### 2.1 Study design

This study employed an explanatory sequential mixed-methods design to examine the relationship between post-traumatic stress (PTS) and post-traumatic growth (PTG) among divorced individuals and to explore the underlying mechanisms of the quantitative findings. In this design, quantitative data were collected and analyzed in the first phase, followed by qualitative data collection to further explain and elaborate the initial results. This approach was selected to enable a more comprehensive understanding of the relationship by integrating statistical patterns with participants’ lived experiences.

### 2.2 Participants

The main sample consisted of 489 divorced individuals (266 women, 223 men) aged 18 years and older residing in Türkiye. Quantitative data were collected from divorced individuals residing in different regions of Türkiye; however, due to convenience sampling, the sample was relatively concentrated in Samsun and surrounding provinces. In the quantitative phase, convenience sampling was used due to its practicality and accessibility in reaching participants.

In the qualitative phase, criterion sampling was employed to select participants based on their levels of post-traumatic stress (PTS) and post-traumatic growth (PTG) identified in the quantitative phase. This approach enabled an in-depth exploration of the research problem by focusing on participants with distinct profiles. Demographic characteristics of the sample are presented in Table 1.

**Table 1:** Demographic characteristics of the study sample.

Variables		*n*	%
Gender	Women	266	54.4
	Men	223	45.6
Age	20–29 years	49	10.0
	30–39 years	145	29.7
	40–49 years	170	34.8
	50 years and above	125	25.6
Income level	Low	37	7.6
	Medium	283	57.9
	High	169	34.6
Education level	High school	143	29.2
	Bachelor’s degree	264	54.0
	Graduate degree	82	16.8
Parenthood	Yes	430	87.9
	No	59	12.1
Duration of marriage	0–5 years	182	37.2
	6–10 years	149	30.5
	11–15 years	78	16.0
	16–20 years	47	9.6
	21 years and above	33	6.7
Time since divorce	0–5 years	304	62.2
	6–10 years	104	21.3
	11–15 years	41	8.4
	16 years and above	39	8.0
Psychological support	Yes	77	15.7
	No	412	84.3

*N* = 489.

In the second phase of the study, four subgroups were formed from the main sample, and four participants from each group were invited (*n* = 16). Participant selection for the qualitative phase was based on cut-off scores and criterion-based grouping. Specifically, participants’ post-traumatic stress (PTS) and post-traumatic growth (PTG) scores from the quantitative dataset were used.

To distinguish extreme groups, participants were classified into “low” and “high” levels based on the upper and lower 27% of the score distributions. Accordingly, four groups were created based on combinations of PTS and PTG levels: high PTS–high PTG, high PTS–low PTG, low PTS–high PTG, and low PTS–low PTG. Participants were then selected from each group. Demographic characteristics of the qualitative subsample are presented in Table 2.

**Table 2:** Demographic characteristics of the qualitative subsample.

Number	Age	Income level	Education level	Number of children	Marriage duration	Time since divorce	Psychological support	Group number
1**	36	High	Bachelor’s	1	7 years	2 years	Yes	1
2*	53	High	Graduate	3	14 years	12 years	No	1
3**	44	Medium	High school	2	9 years	16 years	Yes	1
4*	57	Low	High school	4	24 years	5 years	No	1
5**	32	Medium	Graduate	0	2 years	2 years	No	2
6**	55	High	High school	3	12 years	17 years	No	2
7*	39	Medium	High school	1	4 years	3 years	Yes	2
8*	29	Medium	Bachelor’s	2	5 years	1 year	No	2
9**	46	Medium	Graduate	2	8 years	4 years	No	3
10**	43	Medium	Bachelor’s	2	11 years	5 years	Yes	3
11*	32	Low	Bachelor’s	0	3 years	2 years	No	3
12*	52	High	High school	3	13 years	10 years	No	3
13*	42	High	Bachelor’s	1	7 years	3 years	No	4
14**	37	Medium	Bachelor’s	2	6 years	1 year	No	4
15*	34	Low	Graduate	0	3 years	3 years	Yes	4
16**	49	High	Bachelor’s	1	10 years	4 years	No	4

*N* = 16. **Women; *Men. Group 1 = high PTS–high PTG; Group 2 = high PTS–low PTG; Group 3 = low PTS–high PTG; Group 4 = low PTS–low PTG.

### 2.3 Measures

#### 2.3.1 Demographic information form

The demographic information form was developed by the researchers to obtain descriptive data about the participants. It was designed to support a comprehensive evaluation of the findings and to better capture variations in post-traumatic growth (PTG) after divorce among divorced individuals. The form included questions on age, income level, education level, number of children, duration of marriage, time since divorce, and receipt of psychological support related to divorce.

#### 2.3.2 PTSD checklist for DSM-5 (PCL-5)

The PTSD Checklist for DSM-5 (PCL-5), developed by Weathers et al. (2013) and adapted into Turkish by Boysan et al. (2017), was used to assess participants’ post-traumatic stress levels. In the present study, participants were instructed to respond to the PCL-5 items by considering their divorce-related experiences and the distress associated with the divorce process. The scale consists of 20 items rated on a 5-point Likert scale and includes four subdimensions: re-experiencing, avoidance, negative alterations in cognition and mood, and hyperarousal. Higher scores indicate greater levels of post-traumatic stress. The internal consistency of the scale was high, with a Cronbach’s alpha of 0.94 in the original study, 0.92 in the Turkish adaptation, and 0.94 in the present study.

#### 2.3.3 Posttraumatic growth inventory (PTGI)

The Posttraumatic Growth Inventory (PTGI), developed by Tedeschi and Calhoun (1996) and adapted into Turkish by Kağan et al. (2012), was used to assess participants’ levels of post-traumatic growth. The scale consists of 21 items rated on a 6-point Likert scale. While the original version includes five subdimensions, the Turkish adaptation identified a three-factor structure: changes in self-perception, changes in life philosophy, and changes in relationships. Higher scores indicate greater post-traumatic growth. The scale demonstrated high internal consistency, with Cronbach’s alpha coefficients of 0.90 in the original study, 0.92 in the Turkish adaptation, and 0.93 in the present study.

#### 2.3.4 Semi-structured interview form

In this study, a semi-structured interview form was used to explore participants’ experiences in depth and to allow them to express their perspectives in their own words (Berg and Lune, 2019; Yıldırım and Şimşek, 2018). The interview form was developed by the researcher based on a review of the relevant literature and aimed to examine divorced individuals’ post-divorce experiences, the challenges they encountered, and the factors facilitating or hindering post-traumatic growth. The interviews began with warm-up questions designed to capture participants’ general experiences following divorce. This was followed by open-ended questions addressing changes experienced after divorce, factors influencing the adjustment process, the role of social context, coping strategies, and the impact of this experience on participants’ lives. When necessary, probing questions were used to elicit more detailed responses. Overall, the semi-structured interview form enabled a deeper understanding of the experiences of different participant profiles identified in the quantitative phase and contributed to revealing the psychosocial mechanisms underlying post-traumatic growth after divorce.

### 2.4 Procedure

This study was conducted in two sequential phases following an explanatory sequential mixed-methods design. Prior to data collection, ethical approval was obtained from the Social and Human Sciences Research Ethics Committee of Ondokuz Mayıs University (Decision No: 2025-576; April 25, 2025). In this study, the Turkish adapted versions of the measurement instruments were used. The PTSD Checklist for DSM-5 (PCL-5) and the Posttraumatic Growth Inventory (PTGI) were administered in their Turkish forms, and all necessary ethical and academic permissions for their use were obtained. Before completing the PCL-5, participants were explicitly asked to consider their divorce-related experiences when responding to the items.

In the first phase, quantitative data were collected from 489 participants. In the second phase, a qualitative subsample of 16 participants was formed based on the quantitative findings. Participants were classified according to their post-traumatic stress (PTS) and post-traumatic growth (PTG) scores, and four distinct profiles were formed for the qualitative phase. Participants selected through criterion sampling were interviewed using a semi-structured format. The interviews were transcribed verbatim and analyzed using thematic analysis.

At all stages of the study, participants were informed about the purpose and scope of the research. Participation was voluntary, and informed consent was obtained from all participants. All data were used solely for scientific purposes, and confidentiality and anonymity were strictly maintained.

### 2.5 Data analysis

A total of 491 responses were entered into SPSS 26 for analysis. The dataset was screened for missing values and outliers, and two cases were excluded. The normality of the variables was evaluated using skewness and kurtosis values, as well as visual inspection of histograms. Although the Kolmogorov–Smirnov test was significant, skewness and kurtosis values fell within the acceptable range (±2), and the distributions approximated normality.

To examine the relationship between post-traumatic stress (PTS) and post-traumatic growth (PTG), Pearson correlation analysis and simple linear regression analysis were conducted. Differences in PTG across demographic variables were analyzed using independent samples t-tests and one-way ANOVA, depending on the structure of the variables.

For the qualitative phase, participants were selected based on their PTS and PTG scores using criterion-based grouping, resulting in four distinct profiles. The qualitative data were analyzed using thematic analysis. Interview recordings were transcribed verbatim and repeatedly reviewed to identify meaningful units. These units were coded, and similar codes were grouped into categories, which were then organized into overarching themes. Finally, quantitative and qualitative findings were integrated to provide a comprehensive understanding of the research problem.

## 3 Results

This section first presents the quantitative findings, including descriptive statistics, differences in post-traumatic growth (PTG) across demographic variables, the relationship between post-traumatic stress (PTS) and PTG, and the statistical prediction of PTG by PTS.

### 3.1 Quantitative results

As shown in Table 3, the mean scores for post-traumatic stress (PTS) (*M* = 43.06, SD = 19.22) and post-traumatic growth (PTG) (*M* = 59.49, SD = 20.23) are presented. The wide range of minimum and maximum values, along with relatively high standard deviations, indicates substantial variability among participants. This variability provided a basis for forming distinct participant profiles for the qualitative phase.

**Table 3:** Descriptive statistics for study variables.

Variable	*n*	*M*	SD	Variance	Min	Max
PTS	489	43.06	19.22	369.49	1.00	80.00
PTG	489	59.49	20.23	409.61	8.00	103.00

Differences in post-traumatic growth (PTG) by age were examined using one-way ANOVA, and the results are presented in Table 4.

**Table 4:** One-way ANOVA results for post-traumatic growth (PTG) by age group.

Variable	Age group	*n*	*M*	SD	df	*F*	*p*	*η* ^2^	Post hoc
PTG	20–29 years (1)	49	53.26	15.57	3	4.713	0.003	0.03	4
	30–39 years (2)	145	56.26	18.81					4
	40–49 years (3)	170	61.41	21.68					–
	50 years and above (4)	125	63.09	20.51					1, 2

*N* = 489.

As shown in Table 4, post-traumatic growth (PTG) differed significantly across age groups, *F*(3, 485) = 4.71, *p* = 0.003, *η*^2^ = 0.03. *Post hoc* analyses using Tukey’s HSD test indicated that individuals aged 50 years and above reported significantly higher PTG levels than those aged 20–29 and 30–39.

Differences in post-traumatic growth (PTG) by parenthood status were examined using an independent samples t-test, and the results are presented in Table 5.

**Table 5:** Independent samples t-test results for post-traumatic growth (PTG) by parenthood status.

Variable	Parenthood status	*n*	*M*	SD	t-test			
					*t*	df	*p*	Cohen’s *d*
PTG	No	59	55.98	16.75	−1.667	84.17	0.099	−0.21
	Yes	430	59.98	20.64				

*N* = 489.

As shown in Table 5, post-traumatic growth (PTG) did not differ significantly by parenthood status, *t*(84.17) = −1.67, *p* = 0.099, *d* = −0.21. Although individuals with children reported slightly higher mean PTG scores than those without children, this difference was not statistically significant.

Differences in post-traumatic growth (PTG) by duration of marriage were examined using one-way ANOVA, and the results are presented in Table 6.

**Table 6:** One-way ANOVA results for post-traumatic growth (PTG) by duration of marriage.

Variable	Duration of marriage	*n*	*M*	SD	df	*F*	*p*	*η* ^2^	Post hoc
PTG	0–5 years (1)	182	54.08	16.76	4	5.409	<0.001	0.04	1, 2, 3, 4
	6–10 years (2)	149	62.79	21.65					1
	11–15 years (3)	78	62.00	23.31					1
	16–20 years (4)	47	63.04	20.86					1
	21 years and above (5)	33	63.48	16.93					1

*N* = 489.

As shown in Table 6, post-traumatic growth (PTG) differed significantly across duration of marriage groups, *F*(4, 484) = 5.41, *p* < 0.001, *η*^2^ = 0.04. Post hoc analyses using Tukey’s HSD test indicated that individuals who had been married for 0–5 years reported significantly lower PTG levels than those in all other groups. These findings suggest that longer marital duration may be associated with higher levels of post-traumatic growth.

Differences in post-traumatic growth (PTG) by psychological help-seeking status after divorce were examined using an independent samples *t*-test, and the results are presented in Table 7.

**Table 7:** Independent samples t-test results for post-traumatic growth (PTG) by psychological help-seeking status.

Variable	Psychological help-seeking	*n*	*M*	SD	t-test			
					*t*	df	*p*	Cohen’s *d*
PTG	Yes	77	60.58	16.50	0.601	126.17	0.549	0.07
	No	412	59.29	20.87				

*N* = 489.

As shown in Table 7, post-traumatic growth (PTG) did not differ significantly by psychological help-seeking status after divorce, *t*(126.17) = 0.60, *p* = 0.549, *d* = 0.07. Although individuals who received psychological help reported slightly higher mean PTG scores than those who did not, this difference was not statistically significant.

The results also indicated that PTG did not differ significantly by gender, *t*(487) = −0.88, *p* = 0.378, *d* = −0.08; income level, *F*(2, 486) = 0.06, *p* = 0.943, *η*^2^ < 0.001; or education level, *F*(2, 486) = 0.07, *p* = 0.937, *η*^2^ < 0.001. Since the homogeneity of variance assumption was violated for time since divorce, Welch’s test was considered, and the difference was also nonsignificant, Welch’s *F*(3, 101.65) = 1.89, *p* = 0.136.

To examine the relationship between post-traumatic stress and post-traumatic growth, a Pearson correlation analysis was conducted, and the results are presented in Table 8.

**Table 8:** Correlation between post-traumatic stress (PTS) and post-traumatic growth (PTG).

Variable	PTS	PTG
PTS	1	
PTG	−0.32*	1

*N* = 489. *p < 0.001.

As shown in Table 8, there was a significant negative and moderate correlation between post-traumatic stress (PTS) and post-traumatic growth (PTG), *r* = −0.32, *p* < 0.001. This finding indicates that higher levels of post-traumatic stress are associated with lower levels of post-traumatic growth.

Finally, a simple linear regression analysis was conducted to examine the extent to which post-traumatic stress statistically predicted post-traumatic growth, and the results are presented in Table 9.

**Table 9:** Linear regression analysis predicting PTG from PTS.

Variable	*B*	SE	*β*	*t*	*p*
Constant	78.08	2.13	–	34.78	<0.001
PTS	−0.33	0.04	−0.32	−7.49	<0.001

*N* = 489. *p* < 0.001.

As shown in Table 9, the simple linear regression model was significant, *F*(1, 487) = 56.17, *p* < 0.001. Post-traumatic stress (PTS) was a significant negative statistical predictor of post-traumatic growth (PTG) (*β* = −0.32, *t* = −7.49, *p* < 0.001). In addition, PTS accounted for approximately 10% of the variance in PTG (*R*^2^ = 0.10).

The Durbin–Watson statistic (1.41) indicated no serious autocorrelation issues. Overall, these findings suggest that higher levels of post-traumatic stress are associated with lower levels of post-traumatic growth. Given the cross-sectional design, this result should be interpreted as a statistical association rather than evidence of a causal relationship.

### 3.2 Qualitative results

In the qualitative phase of the study, semi-structured interviews were conducted to explore the dynamics related to post-traumatic growth after divorce. The data were analyzed using thematic analysis, and the findings are presented under two main categories: factors facilitating and factors hindering post-traumatic growth.

The findings are presented comparatively based on participant groups formed according to their levels of post-traumatic stress (PTS) and post-traumatic growth (PTG).

The themes, categories, and codes related to factors facilitating post-traumatic growth are presented in Table 10. These findings were derived from the thematic analysis of responses to open-ended questions exploring participants’ post-divorce experiences.

**Table 10:** Qualitative analysis of factors facilitating post-traumatic growth after divorce.

Themes	Categories	Codes	Group			
			1	2	3	4
Future orientation	Hope	Belief in Starting Over	3/4	1/4	4/4	2/4
	Setting New Goals	Ability to Set New Goals	3/4	0/4	3/4	2/4
Spiritual resources	Religious Practices/Worship	Praying	4/4	3/4	4/4	1/4
	Spiritual Coping	Meaning-Making	2/4	1/4	3/4	1/4
Personal strengths	Resilience	Belief in Staying Strong	4/4	1/4	4/4	2/4
	Self-awareness	Self-recognition	2/4	2/4	3/4	2/4
	Cognitive Restructuring	Reappraisal of the Event	3/4	1/4	4/4	2/4
Social resources	Family Support	Focusing on Family of Origin	4/4	3/4	3/4	2/4
	Support from Children	Devotion to One’s Child	3/4	2/4	3/4	1/4
	Friend Support	Spending Time with Friends	3/4	2/4	2/4	1/4
Life conditions	Economic Independence	Having One’s Own Income	2/4	0/4	2/4	1/4
	Relocation	Changing Residence	3/4	2/4	3/4	0/4

Group 1 = high PTS–high PTG; Group 2 = high PTS–low PTG; Group 3 = low PTS–high PTG; Group 4 = low PTS–low PTG. PTS = post-traumatic stress; PTG = post-traumatic growth.

The qualitative findings suggested that factors facilitating post-traumatic growth after divorce clustered under multiple themes, including future orientation, spiritual resources, personal strengths, social resources, and life conditions. These themes reflect participants’ lived experiences during the post-trauma process and illustrate the multidimensional nature of growth.

The theme of future orientation, encompassing hope and setting new goals, was particularly prominent among participants with low post-traumatic stress (PTS) and high post-traumatic growth (PTG). Participants in this group tended to reconstruct their lives and develop new purposes following divorce. For instance, one participant stated, *“After the divorce, I thought my life was over, but over time I found new goals. I started and completed my master’s degree, and now I am preparing for a PhD”* (P9). Another participant emphasized the role of hope by stating, *“My belief that things would get better kept me going, and I was able to stand up again”* (P12).

The theme of spiritual resources, including religious practices and spiritual coping, similarly emerged more strongly in groups characterized by higher PTG. Participants frequently referred to prayer and meaning-making as central strategies for coping with the post-divorce process. One participant expressed this as *“The prayers I made while I was at my lowest helped me find my way out”* (P11), while another noted, *“When I evaluated this process through my spiritual beliefs, I was able to respond more calmly to what I experienced”* (P3).

The theme of personal strengths, comprising resilience, self-awareness, and cognitive restructuring, was particularly salient among participants with higher PTG levels. These individuals tended to frame divorce not only as a negative life event but also as an opportunity for self-discovery and personal growth. Cognitive reappraisal and the belief in remaining strong appeared especially influential. One participant stated, *“What I went through broke me, but it also helped me understand myself better”* (P2), while another noted, *“At first it was very difficult, but over time I learned to see this process from a different perspective”* (P10). These findings suggest that internal psychological resources may play an important role in facilitating growth (Table 11).

**Table 11:** Qualitative analysis of factors hindering post-traumatic growth after divorce.

Themes	Categories	Codes	Group			
			1	2	3	4
Cognitive barriers	Rumination	Recurrent Thoughts About the Past	1/4	4/4	0/4	3/4
	Difficulty in Acceptance	Inability to Mentally Let Go	1/4	3/4	1/4	2/4
Economic barriers	Uncertainty	Future Anxiety	1/4	3/4	1/4	2/4
	Financial Difficulties	Financial Strain	2/4	3/4	2/4	3/4
Relational barriers	Lack of Support	Perceived Abandonment	2/4	4/4	2/4	3/4
	Loss of Trust	Inability to Trust Others	1/4	2/4	1/4	2/4
Relational barriers	Negative Emotions	Anger and Sadness	2/4	4/4	0/4	3/4
	Hopelessness	Negative Expectations to Future	0/4	3/4	1/4	4/4
	Self-blame	Guilt and Regret	1/4	4/4	0/4	2/4
Social barriers	Stigmatization	Divorcee Label	1/4	2/4	0/4	2/4
	Social Judgment	Negative Social Perceptions	2/4	4/4	1/4	4/4
	Family Pressure	Family Interference	1/4	3/4	1/4	3/4

Group 1 = high PTS–high PTG; Group 2 = high PTS–low PTG; Group 3 = low PTS–high PTG; Group 4 = low PTS–low PTG. PTS = post-traumatic stress; PTG = post-traumatic growth.

The theme of social resources, including family support, support from children, and friendships, was also more pronounced among participants with higher PTG. Social support played a crucial role in helping individuals recover and rebuild their lives after divorce. Family support, in particular, was frequently described as a primary source of emotional strength, while children served as a strong motivational factor for some participants. For example, one participant stated, *“If it were not for my family, I would not have gotten through this process so easily; they were my biggest support”* (P1), whereas another noted, *“I had to be strong for my child, and that gave me the strength to keep going”* (P12). Friend support was also mentioned but appeared less central compared to family support. These findings suggest that social support may function as both a protective and strengthening factor in the growth process.

Finally, the theme of life conditions, including economic independence and relocation, emerged as another important facilitator, particularly among participants with higher PTG. Participants emphasized that being financially independent and making changes to their living environments contributed to restructuring their lives. Economic independence, in particular, was associated with a greater sense of control and psychological wellbeing. One participant stated, *“After I started earning my own money, I felt stronger; I was no longer dependent on anyone”* (P5), while another expressed, *“Changing my home felt like a new beginning; it was as if I reset my life”* (P14). These findings suggest that structural changes in life conditions may serve as external facilitators of post-traumatic growth.

The qualitative findings suggested that factors hindering post-traumatic growth after divorce were structured around cognitive, economic, relational, emotional, and social barriers.

When future orientation was considered in terms of hope and expectations about the future, it appeared weaker particularly among participants with high post-traumatic stress (PTS) and low post-traumatic growth (PTG). Participants in this group tended to develop more pessimistic and uncertainty-focused perspectives regarding their post-divorce lives. For example, one participant stated, *“I cannot make any plans for the future; everything feels uncertain”* (P6), while another noted, *“No matter what I did, I thought my life would never improve”* (P8). In contrast, participants with higher PTG expressed more positive expectations about the future. These findings suggest that diminished hope and goal orientation may hinder the growth process.

The theme of economic barriers, including financial difficulties and uncertainty, was more prominent among participants with lower PTG. Participants frequently emphasized that income loss and financial strain following divorce made the adjustment process more challenging. One participant stated, *“After the divorce, I struggled financially; managing on my own was exhausting”* (P15), while another expressed, *“My biggest concern about the future was my financial situation; I constantly felt stressed about it”* (P8). In contrast, participants who were more economically independent appeared to cope more effectively. These findings suggest that economic conditions may function as an external constraint on post-traumatic growth.

Relational barriers, including lack of support and loss of trust, also emerged as important obstacles, particularly among participants with lower PTG. Participants described feelings of loneliness and difficulties trusting others after divorce. For instance, one participant stated, *“No one was there for me during this process; I felt completely alone”* (P5), while another noted, *“I lost my trust in people, so I could not get close to anyone”* (P13). These findings suggest that relational disconnection may limit the growth process.

Emotional barriers, particularly negative emotions and self-blame, were more evident among participants with high PTS and low PTG. Participants’ narratives reflected intense sadness and guilt, which appeared to complicate their adjustment. One participant stated, *“After the divorce, I constantly felt sad”* (P6), while another expressed, *“I felt as if everything was my fault”* (P7). These findings suggest that persistent negative emotional states may inhibit post-traumatic growth.

Overall, these findings suggest that post-traumatic growth after divorce may be constrained by a combination of internal (cognitive and emotional) and external (economic and relational) barriers that interfere with adaptive coping and meaning-making processes.

## 4 Discussion

The present study aimed to examine the relationship between post-traumatic stress (PTS) and post-traumatic growth (PTG) in divorced individuals and to identify the factors related to this relationship through a mixed-methods approach. The findings provide important insights into both individual and contextual mechanisms underlying post-traumatic growth after divorce.

One of the primary findings of the study is that PTG levels differed significantly across age groups, with older participants reporting higher PTG levels. This pattern may be related to the accumulation of life experiences, the development of coping skills, and an enhanced ability to interpret stressful events from a broader perspective among older individuals (D'Zurilla et al., 1998; Lee and Pollack, 1978). Consistent with this interpretation, previous studies have also reported a positive association between age and PTG (Boyle et al., 2017; Patrick and Henrie, 2016). These findings suggest that younger individuals may require additional support in navigating post-divorce adjustment, highlighting the potential value of psychoeducational interventions tailored to this group.

Another finding of the study is that PTG did not differ significantly by parenthood status. Although participants with children reported slightly higher mean PTG scores than those without children, this difference was not statistically significant. This finding suggests that parenthood status alone may not be sufficient to explain post-traumatic growth after divorce. Rather, the role of parenthood may depend on additional factors such as parenting responsibilities, custody arrangements, social support, economic resources, and the quality of parent–child relationships. Therefore, future studies should examine parenthood in a more detailed manner by considering not only whether individuals have children, but also the nature of parenting-related responsibilities and support systems after divorce.

The findings further indicate that individuals who divorced within the first 5 years of marriage reported lower PTG levels compared to those with longer marital durations. This may be related to feelings of failure associated with early marital dissolution, which can negatively influence self-perception and post-divorce adjustment (Apostu, 2019; Perrig-Chiello et al., 2015; Markman et al., 2010). Such perceptions may persist beyond the divorce itself, potentially limiting opportunities for growth.

In addition, PTG did not differ significantly according to whether participants received psychological help after divorce. Although participants who received psychological help reported slightly higher mean PTG scores than those who did not, this difference was not statistically significant. This finding suggests that receiving psychological help alone may not be sufficient to explain differences in PTG. The contribution of psychological support to post-divorce growth may depend on several factors, such as the timing, duration, quality, and type of support received, as well as individuals’ readiness to engage in meaning-making and cognitive restructuring processes. Therefore, future studies should examine psychological help-seeking in greater detail by considering the characteristics and perceived effectiveness of professional support.

Finally, the study found a significant negative relationship between PTS and PTG, with PTS also emerging as a significant negative statistical predictor of PTG. These findings suggest that higher levels of traumatic stress are associated with lower levels of growth-related processes. However, it should be noted that PTS explained only 10% of the variance in PTG. This indicates that although PTS is significantly associated with PTG, it is not sufficient on its own to explain post-traumatic growth after divorce. The remaining unexplained variance suggests that other individual, relational, and contextual factors may play an important role in growth-related processes. This interpretation is supported by the qualitative findings, which showed that hope, social support, spiritual coping, cognitive restructuring, economic independence, and social pressure were related to participants’ growth experiences.

This relationship can be understood in terms of cognitive and emotional resource depletion, where elevated stress may be associated with difficulties in individuals’ ability to process and reframe their experiences (Schweizer and Dalgleish, 2011; McNally, 2006; Hobfoll, 1991). In other words, when stress exceeds a certain threshold, individuals may prioritize coping and stabilization rather than growth (Koncz et al., 2024). Moreover, the relationship between PTS and PTG may not be strictly linear; moderate levels of stress may be associated with growth, whereas excessive stress may be linked to lower growth (Marziliano et al., 2020; Ochoa et al., 2017; Jin et al., 2014). Future research should therefore consider potential mediating and moderating variables that may clarify the mechanisms underlying this relationship.

### 4.1 Dynamics influencing post-traumatic growth after divorce

While the quantitative findings revealed that post-traumatic growth (PTG) varies across specific demographic and psychological variables, the qualitative findings provide deeper insight into the underlying processes and experiential dynamics shaping these differences.

First, the factors facilitating post-traumatic growth after divorce appear to align with the core mechanisms proposed in the PTG literature. PTG is closely associated with the disruption of core belief systems and the subsequent reconstruction of meaning through cognitive processing (Kau et al., 2024). In this context, future-oriented dynamics such as hope and setting new goals appear to be associated with individuals’ efforts to reorganize their experiences and rebuild a sense of direction after divorce (Confino et al., 2023; Umer and Elliot, 2021). These findings suggest that growth is not a passive recovery process but rather an active reconstruction of one’s life narrative.

The prominence of social support and interpersonal resources is also noteworthy. Existing research indicates that social support plays a critical role in the relationship between post-traumatic stress (PTS) and PTG (Altınışık and Şanlı, 2024; Bryngeirsdottir and Halldorsdottir, 2022; Calhoun et al., 2022; Zhao et al., 2020). Social support may facilitate emotional expression, promote psychological relief, and enable individuals to generate new meanings from their experiences (Donovan, 2022; Kaniasty, 2012). From this perspective, the emergence of social resources in the findings suggests that growth is not solely an individual process but may also be embedded within relational contexts (Sippel et al., 2015).

Similarly, the role of spiritual resources appears to point to the meaning-making dimension of PTG. Research suggests that spirituality can mediate the relationship between PTS and PTG and is associated with higher levels of growth (Feng et al., 2024; Askay and Magyar-Russell, 2009). Spiritual coping may be associated with individuals’ efforts to reinterpret their experiences within a broader existential framework, transforming the question from “Why did this happen to me?” to “What has this experience brought into my life?” This transformation may represent a critical threshold in the emergence of post-traumatic growth after divorce (Poljak Lukek and Valenta, 2024).

In addition, findings related to personal strengths—particularly cognitive restructuring and self-awareness—highlight the central role of cognitive processes in PTG (De Castella and Simmonds, 2013). Prior research has identified cognitive reappraisal as one of the strongest predictors of growth (Cadell et al., 2003; Tedeschi and Calhoun, 1996). Thus, the individual’s ability to process and reinterpret the experience in an adaptive manner appears to be closely associated with growth-related processes. Conversely, when this process takes the form of maladaptive rumination, it may hinder growth (Allen et al., 2022).

An important finding of this study is that these facilitating factors may not only support growth but also appear to be associated with buffering processes in the context of post-traumatic stress. In other words, PTG cannot be explained solely by lower levels of stress; rather, individuals’ psychosocial resources may be linked to how stress is experienced and how growth-related processes emerge. This suggests that post-traumatic growth after divorce is not a linear outcome but a dynamic process emerging from the interaction between stress levels and coping resources (Henson et al., 2021; Amato and Hohmann-Marriott, 2007).

When the processes that hinder post-traumatic growth after divorce are examined, the identified themes appear to cluster primarily around cognitive, emotional, and relational domains, closely aligning with core mechanisms known to constrain post-traumatic growth (PTG).

Cognitive barriers—particularly rumination and the inability to mentally disengage from the experience—were more pronounced among individuals with higher levels of post-traumatic stress (PTS). This finding suggests that repetitive cognitive engagement with the traumatic experience may be associated with difficulties in adaptive meaning-making by keeping individuals psychologically anchored to the event. Consistent with prior research, maladaptive (intrusive) rumination has been associated with lower PTG, whereas more deliberate and structured cognitive processing has been associated with higher growth (Allen et al., 2022; Stockton et al., 2011).

Economic barriers, which were more evident among participants with high PTS and low PTG, suggest that growth may not be solely a psychological phenomenon but may also be shaped by structural conditions. Financial strain and economic uncertainty may be associated with a reduced sense of security and fewer psychological resources, and thereby restrict the cognitive and emotional capacities required for growth (Collins, 2025; Bryngeirsdottir and Halldorsdottir, 2022).

Similarly, relational barriers—such as lack of support and diminished trust—were more prominent among individuals experiencing higher stress levels, suggesting that weakened social bonds may hinder growth processes. Given that social support is widely recognized as a protective factor in PTG, its absence may be associated with risk factors that limit growth (Calhoun et al., 2022).

From an emotional standpoint, intense negative emotions including anger, sadness, guilt, and hopelessness were more frequently reported in high-PTS groups. These emotional states may be associated with difficulties in cognitive restructuring processes, including reduced psychological flexibility and difficulties in reinterpreting experiences (Confino et al., 2023). In particular, the prominence of hopelessness among low-PTG participants suggests that the inability to construct positive future expectations may be associated with barriers to growth.

Finally, social barriers—such as stigma, societal judgment, and family pressure—were also more pronounced among individuals with elevated stress levels. This finding highlights that divorce is not merely an individual psychological experience but also a socially embedded process, in which external evaluations and cultural expectations may be associated with coping trajectories (Apak and Çalıcı, 2026).

Overall, these findings suggest that post-traumatic growth after divorce may be associated not only with individuals’ internal resources but also with the broader social context in which they are embedded. In this respect, growth-related processes may be constrained when cognitive overload, emotional distress, relational disconnection, and contextual pressures converge, limiting individuals’ capacity to engage in adaptive meaning-making processes.

### 4.2 Integration of quantitative and qualitative findings

The integration of the quantitative and qualitative findings provides a more comprehensive understanding of post-traumatic growth after divorce. Quantitatively, PTG was negatively associated with PTS, and PTS statistically predicted PTG; however, the regression model explained only 10% of the variance. This limited explanatory power suggests that PTG cannot be understood solely through stress levels. The qualitative findings help explain this result by showing that growth-related experiences were also associated with hope, goal setting, cognitive restructuring, spiritual coping, social support, economic independence, and relocation. Similarly, the quantitative finding that PTG differed significantly by age and duration of marriage was supported by qualitative narratives emphasizing life experience, future orientation, resilience, and meaning-making. In contrast, the absence of significant differences by parenthood status and psychological help-seeking suggests that these variables alone may not explain growth; the qualitative findings further indicate that the quality of support, parenting responsibilities, economic resources, and personal readiness for meaning-making may be more important than the mere presence of children or receipt of psychological help. Overall, the integrated findings suggest that post-traumatic growth after divorce is associated with a dynamic interaction between stress, individual coping resources, relational support, and contextual conditions.

## 5 Limitations

Although the quantitative data were collected from participants residing in different regions of Türkiye, the sample was relatively concentrated in Samsun and surrounding provinces due to the use of convenience sampling. Therefore, the findings should not be interpreted as fully representative of the entire divorced population in Türkiye. Future studies may employ stratified or probability-based sampling strategies with more balanced regional representation to enhance generalizability.

Another limitation concerns the qualitative phase. Although criterion sampling allowed for the purposeful selection of participants with distinct PTS–PTG profiles, the qualitative subsample was relatively small, with 16 participants in total and four participants in each group. Therefore, the qualitative findings should be interpreted as exploratory and explanatory rather than representative. Future studies may include larger and more diverse qualitative samples to examine whether similar themes emerge across different divorced populations.

A further limitation is related to the use of self-report measures. Since the quantitative data were collected through self-report instruments, the findings may be affected by common method variance and response biases. In addition, divorce may be a culturally sensitive issue in Türkiye due to stigma, social judgment, and family expectations. Therefore, participants may have underreported or overreported certain experiences because of social desirability concerns. Future studies may reduce this limitation by using multi-method assessment strategies, including clinician-administered measures, longitudinal interviews, or social desirability control measures.

The cross-sectional design of the quantitative phase is another limitation. Because PTS and PTG were measured at a single time point, the findings cannot establish temporal precedence or causal relationships between post-traumatic stress and post-traumatic growth. Therefore, the regression results should be interpreted as indicating statistical prediction rather than causality. Future longitudinal studies are needed to examine how PTS and PTG change over time and how their relationship unfolds throughout the post-divorce adjustment process. Although participants were instructed to respond to the PCL-5 items by focusing on their divorce-related experiences, it is still possible that other stressful or traumatic life events may have influenced their responses.

## 6 Conclusion and implications

This study suggests that post-traumatic growth (PTG) following divorce is associated with a complex interplay between post-traumatic stress (PTS) and psychosocial resources. While higher levels of PTS are associated with lower PTG, qualitative findings suggest that growth-related processes are associated with mechanisms such as cognitive restructuring, social support, spirituality, and future orientation. Conversely, cognitive overload, emotional distress, economic strain, and relational disruptions may be associated with constraints in growth-related processes.

These findings suggest that PTG is not merely related to reduced stress but may be associated with the interaction between stress and available coping resources. From a practical perspective, interventions targeting divorced individuals should focus on enhancing adaptive cognitive processing, strengthening social support systems, and addressing structural barriers such as economic difficulties. In particular, improving access to psychological support services and developing targeted psychoeducational programs may support growth-related processes, although the effectiveness and characteristics of such support should be examined in greater detail in future studies.

From a research perspective, future studies should examine post-traumatic growth after divorce through more comprehensive models that include individual, relational, and contextual variables. Variables such as social support, coping strategies, meaning-making, economic resources, parenting responsibilities, perceived stigma, and access to professional support may help clarify why some individuals experience higher levels of growth whereas others remain more vulnerable after divorce. In addition, longitudinal studies are needed to better understand how PTS and PTG change over time and how growth-related processes unfold during post-divorce adjustment.

From a practice and policy perspective, the findings point to the importance of developing accessible and context-sensitive support services for divorced individuals. Counseling and psychoeducational programs may focus on strengthening adaptive cognitive processing, supporting future-oriented goal setting, enhancing social support, and addressing emotional, economic, and relational barriers. At the policy level, community-based psychosocial support programs, affordable counseling services, and stigma-reduction initiatives may contribute to more supportive post-divorce adjustment conditions.

## Data Availability

The datasets generated and/or analyzed during the current study are not publicly available due to privacy and ethical restrictions but are available from the corresponding author on reasonable request.
